# The Roles of Endstopped and Curvature Tuned Computations in a Hierarchical Representation of 2D Shape

**DOI:** 10.1371/journal.pone.0042058

**Published:** 2012-08-09

**Authors:** Antonio J. Rodríguez-Sánchez, John K. Tsotsos

**Affiliations:** 1 Intelligent and Interactive Systems, University of Innsbruck, Innsbruck, Austria; 2 Centre for Vision Research and Dept. of Computer Science and Engineering, York University, Toronto, Ontario, Canada; Georgia State University, United States of America

## Abstract

That shape is important for perception has been known for almost a thousand years (thanks to Alhazen in 1083) and has been a subject of study ever since by scientists and phylosophers (such as Descartes, Helmholtz or the Gestalt psychologists). Shapes are important object descriptors. If there was any remote doubt regarding the importance of shape, recent experiments have shown that intermediate areas of primate visual cortex such as V2, V4 and TEO are involved in analyzing shape features such as corners and curvatures. The primate brain appears to perform a wide variety of complex tasks by means of simple operations. These operations are applied across several layers of neurons, representing increasingly complex, abstract intermediate processing stages. Recently, new models have attempted to emulate the human visual system. However, the role of intermediate representations in the visual cortex and their importance have not been adequately studied in computational modeling.

This paper proposes a model of shape-selective neurons whose shape-selectivity is achieved through intermediate layers of visual representation not previously fully explored. We hypothesize that hypercomplex - also known as endstopped - neurons play a critical role to achieve shape selectivity and show how shape-selective neurons may be modeled by integrating endstopping and curvature computations. This model - a representational and computational system for the detection of 2-dimensional object silhouettes that we term 2DSIL - provides a highly accurate fit with neural data and replicates responses from neurons in area V4 with an average of 83% accuracy. We successfully test a biologically plausible hypothesis on how to connect early representations based on Gabor or Difference of Gaussian filters and later representations closer to object categories without the need of a learning phase as in most recent models.

## Introduction

Since the foundation of modern neuroanatomy by Ramón y Cajal, who gave a detailed description of the nerve cell organization in the central and peripheral nervous system [1]–[4], great progress has been achieved in understanding the human brain. At the same time, computing power and technology have provided more sophisticated tools to study the brain and its great complexity. Computational neuroscience has appeared as an important methodology for formalizing and testing new hypotheses on how that complex system may perform certain operations.

Over the last decades, many models inspired by advances in the anatomy of the visual cortex have been presented, the earliest from the late 60 s and early 70 s [5]–[8]. A subsequent and very influential model is Fukushima's Neocognitron [9]. The Neocognitron is a self-organizing neural network model that achieves position invariance and later demonstrated to perform well on digit recognition [10]. The network contains an input layer followed by a cascade of *S-cells* (for simple cells) and *C-cells* (complex cells). After unsupervised training thanks to a self-organization process, one of the C-cells in the last layer will respond selectively to the input pattern used in training. Later models, based on Fukushima's foundation, that included backpropagation [11] were also successful at the task of handwriting digit recognition [12], [13].

Since then, there have been several relevant works. Visnet [14] consists of a four layer network that achieves invariant object recognition. The most crucial part of such a method is a trace learning rule that is Hebbian based. To achieve translation invariance, the network is trained with inputs at different positions. Riesenhuber and Poggio's [15]–[19] model consists of five hierarchical levels of *S* and *C* neurons (following Fukushima's Neocognitron [9]) that are connected through linear operations in one layer and non-linear (MAX) in the next (the strongest units determine the response of the system). The first level receives input from the retina and is composed of simple neuron receptive fields that analyze orientations. The next levels account for more complex features (e.g. junctions). The last level is composed of view-tuned neurons that achieve position and scale invariance.

Amit [20], [21] presents a parallel neural network for visual selection. This network is trained to detect candidate locations for object recognition. Objects are represented as composed of features localized at different locations with respect to an object centre. Simple features (edges and conjunctions) are detected in lower levels, while higher levels carry out disjunctions over regions. Suzuki and colleagues [22] construct a model of the form pathway based on predictive coding [23], [24]. Predictive coding hypothesizes that feedback connections from high to lower-order cortical areas carry predictions of lower-level neural activities. Feedforward connections carry residual errors between predictions and the actual lower-level activities. In the model, a fast coarse processing precedes and contrains more detailed processing.

None of the models presented until now fully explore the possible contributions of intermediate representations as they are known in the brain. Common to most models is a first step that performs some sort of edge-detection in a similar way to some V1 neurons in the brain. Even though some of the proposals may include hierarchies with intermediate representations (e.g. [19], [25]), these representations do not include much of the complexity now known to exist in the intermediate layers of the visual cortex. The usual modeling of intermediate layers to date is a simple composition of earlier features to approximate shape without computing curvature or shape directly. Here, we propose a more direct approach, one that provides models of units that compute shape properties directly using several novel neurally-based computations. Distinct from the best of the previous approaches, we do not use simple hierarchical composition of a common neural type but rather, define new neural selectivities for each of several intermediate visual computation layers.

Models up to now have been stagnant on the representation of contours following Marr's [26], [27] primal sketch, that is, edge combinations are used to represent shapes and objects. Models have added layers of *S* and *C* cells following early systems [9] into higher levels of the hierarchy, not considering that cells in those higher levels perform quite different, more complex, operations. There has been some progress on how hypercomplex cells, also known as endstopped, may be defined [28]–[30], but except for the work of [31]–[33] on figure-ground segregation, the role of endstopping has been neglected. Here, following this past work, we hypothesize that endstopped neurons play an important role in encoding curvature and shape.

We present a biologically plausible model for shape representation, 2DSIL, where the focus is on 2D silhouettes. In the following section we describe in detail each layer in the model. Next we show the strongly positive results of testing the model with stimuli used in previous single-cell recording studies followed by a discussion regarding the characteristics of 2DSIL. In a previous paper [34] we showed that even when this representation is used within a recognition system, it outperforms the leading competing models. Material and methods are presented at the end.

## Results

In this section we explain how shape selectivity may be achieved with a model that incorporates intermediate layers inspired by the primate visual system. We demonstrate the performance of our model by comparing computed responses with neurons from area V4.

### Incorporating endstopping and curvature in a model of shape representation

**Figure 1:**
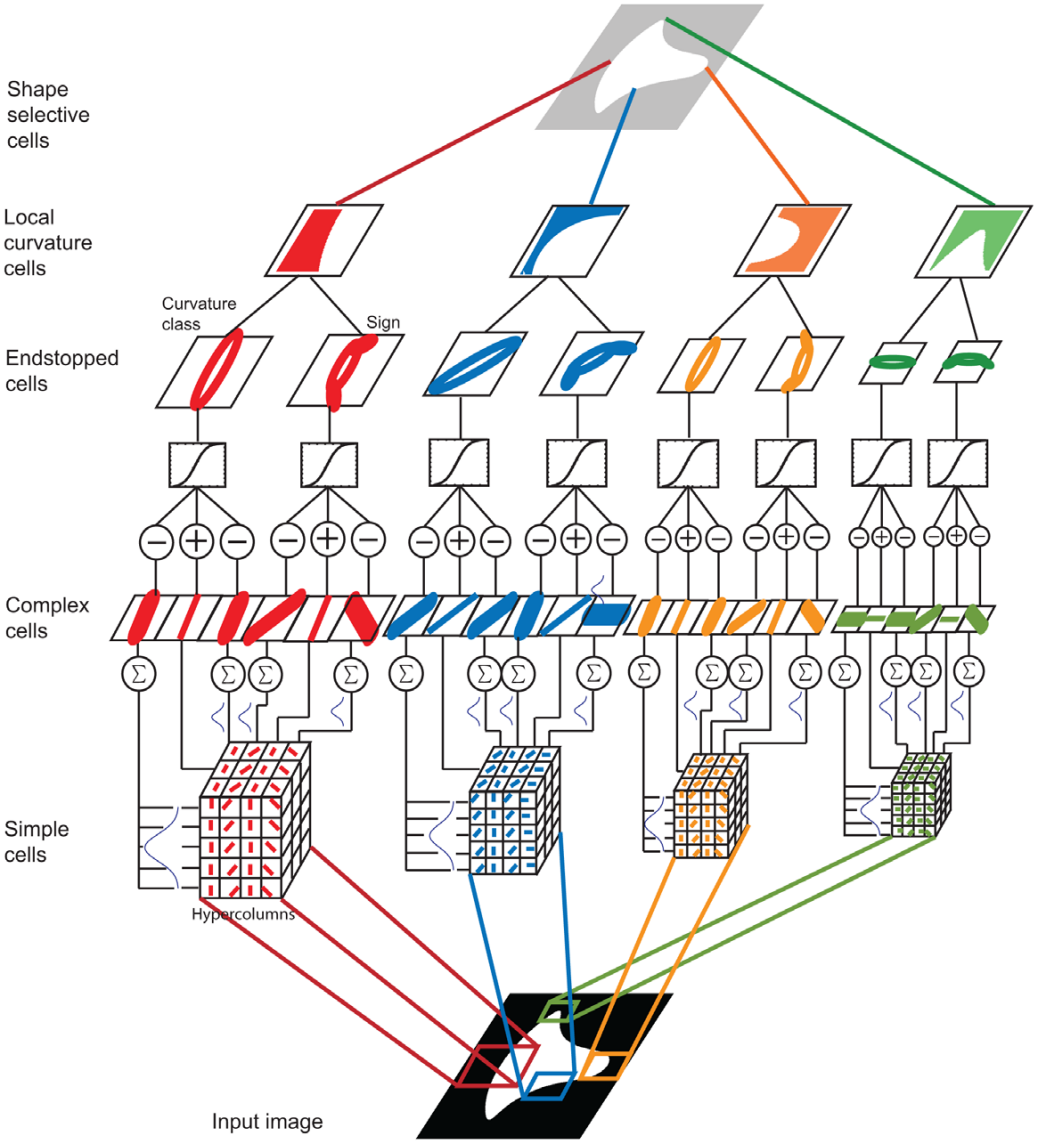
Architecture of the representational and computational system for the detection of 2-dimensional object silhouettes (2DSIL).

Figure 1 presents a depiction of the proposed architecture, which comprises simple, complex, endstopped, local curvature and shape-selective cells that are described next in detail. In what follows whenever a neuron is referred to as model neuron/cell it is one developed for our theory. A neuron or cell referred to without the model adjective is a biological one.

#### Model simple cells

Simple neurons of visual area V1 are sensitive to bar and edge orientations as previous models also stipulate. Common spatial response profiles to model simple neurons in area V1 include Gabor filters [35] and Difference of Gaussians. The latter provides a better fit to neuronal responses [36] and accordingly gave better results in our case than the Gabor filter formulation:


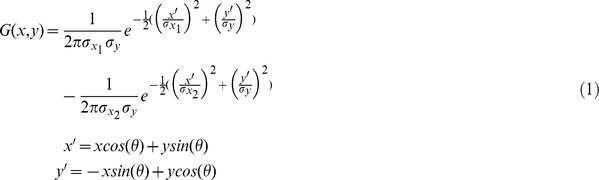


where 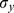 is the height and 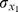 and 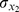 are the width of each Gaussian function. 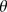 is their orientation. The relation between these parameters may be referred to as the aspect ratio 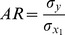 and the width ratio 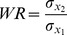. Size of filters were 4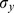. As with all the model neurons within 2DSIL, these are defined at multiple scales, each scale being band-pass for a range of receptive field sizes, with the number of scales represented appropriate for the modelling task. Values assigned to these parameters are exposed in the methods section.

Cells in area V1 are heterogeneous, i.e. they are not all uniform. In the realization of the model, four different groups of simple cells were designed, varying sizes and values of width and length. Model simple cells are organized into hypercolumns. Within a hypercolumn, cells are organized at the same orientation but are spatially displaced and combined into model complex cells as described next (Figure 1), however there is no input from left and right eye since binocular responses are not considered in this study. Model simple cells are at different orientations and scales.

#### Model complex cells

Complex cells have a sensitivity for bars and orientations as well, but their receptive fields are larger than the ones of simple neurons. Hubel and Wiesel [37]–[39] suggested that complex cells may integrate the responses of simple cells. In addition to this, [40] showed that complex cells may be the result of the addition of simple cells along the axis perpendicular to their orientation. Following these studies, in our model, a complex cell is the weighted sum of 5 laterally displaced model simple cells within a column. The model complex cell response is given by [30]:


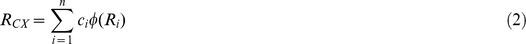



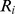
 is the response of the ith cell and 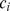 is its weight. Model cells are Gaussian weighted by position, with weight inversely proportional to distance to the center. 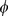 is a rectification function, where any value less than 0 is set to 0. Model simple cells combining into a model complex cell are laterally displaced, their displacement being proportional to the cell's *size* as well as the height (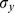) and width (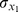) of the Gaussian function. Displacement is in the direction of the orientation perpendicular to the preferred one (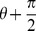, using the *modulo* function to keep values in the range 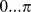) and are given by 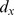 (displacement in *x* axis) and 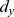 (displacement in *y* axis) in the following equation:


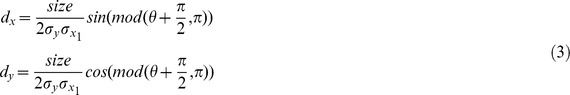


**Figure 2:**
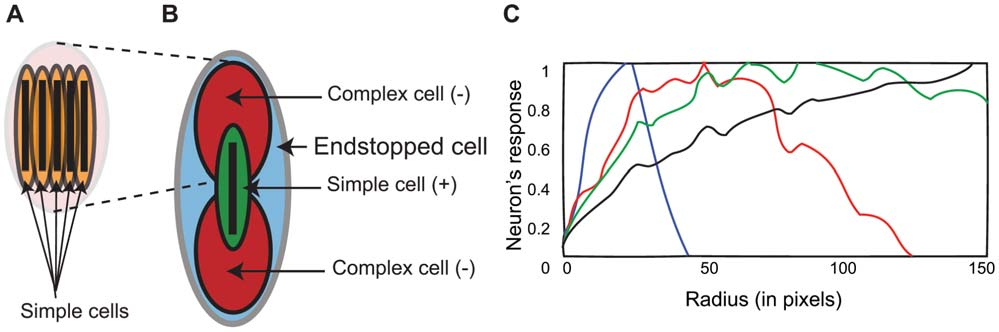
Endstopping. (A) Model complex cell. (B) Structure of model endstopped cell. (C) Response of the model endstopped cells to different radius of curvatures. Simple cell sizes were 40 (blue color), 80 (red color), 100 (green color) and 120 pixels (black color). 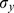 = (10,20,25,30). *AR* (aspect ratio) = (1.15,2,3,4). *WR* (width ratio) = 2.5 for all cells. Gain *c* = (0.7,0.8,1,2). Responses were normalized for the range [0,1].

The construction of a model complex neuron is depicted in Figure 2A. The orientation of its model simple neuronal components in this case is for 90 (vertical), while the 5 model simple cells are organized perpendicularly (spatially displaced but overlapping) to this preferred orientation, that is, 0. This results in slightly less sensitivity for orientations since each model complex cell integrates five model simple cells. A model complex neuron yields a positive response for stimuli at more locations inside its receptive field and their receptive fields are larger as well. These characteristics follow [37]–[39] and up to this point our model simple and complex cells follow [9] and share some similarities with its followers as well [15], [21], [41].

#### Model endstopped cells

Endstopped - also known as hypercomplex - neurons respond to contours, both real and illusory [42]. A more recent study [43] has found that although V2 neurons are mainly selective for angles and corners, these neurons also showed submaximal responses for bars. Model endstopped cells result from the difference between a simple cell and two displaced complex cells [44]. At this point, our model diverges strongly away from formulations in the previous works cited above. When simple and complex cells are combined at the same orientation we can distinguish between degrees of curvature. Through the use of model complex cells at different orientations with respect to the simple cell, we can obtain the sign of the curvature. These two model neuron types are explained next.

#### Model cells discriminant to the degree of curvature

This model endstopped cell is the neural convergence of a model simple neuron and two displaced model complex neurons selective for the same orientation as follows (Figure 2B):


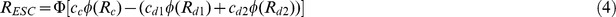



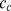
, 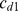 and 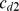 are the gains for the center and displaced cells. 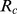, 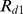 and 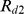 are the responses of the center and the two displaced cells. 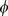 is a rectification function, where any value less than 0 is set to 0. 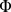 is:


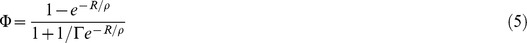


This sigmoidal function - whose parameter values are given in the methods section - scales responses to highly intense stimuli. Displaced cells are shifted 1/2 of their receptive field size in the direction of their prefered orientation. The center simple cell has an excitatory effect while the two complex cells (at the top and bottom in Figure 2B) have an inhibitory effect, which are wider than the center cell, following [45], [46]. This design follows the work of [28], [30], [47] and [44], [45], [48], [49].

Thanks to this configuration of simple and complex cells, we obtain a coarse estimation of curvature such that different curvatures can be discriminated into classes. Figure 2C shows how this type of cell can discriminate among different degrees of curvature. The plot shows how arcs of different radius provide different responses from this type of cell depending on the size of the component simple and complex cells. The scales of the simple and complex neurons that are combined in the configuration of endstopped cells play an important role in this curvature discrimination as it is shown in Figure 2C. Different neuronal sizes provide a different response to different degress of curvature. The model endstopped smallest neuron (Figure 2C blue plot, simple cell size 40 pixels) is selective for very high curvatures, while the largest model enstopped neuron (Figure 2C black plot, simple cell size 120 pixels) was selective to very broad curvatures, in-between scales (sizes of 80 and 100 pixels) provide preferred responses to intermediate curvatures (red and green plots). Note that this configuration also has maximal responses to bars of a specified length (that of the simple cell at the center) as it is the case of real endstopped cells as well. Also note that the choice of these sizes, and even the number of sizes or scales in the model overall, are at the discretion of the modeler so that the space of visual contours addressed by the model are best fit by the scales represented.

#### Model cells selective to the sign of curvature

**Figure 3:**
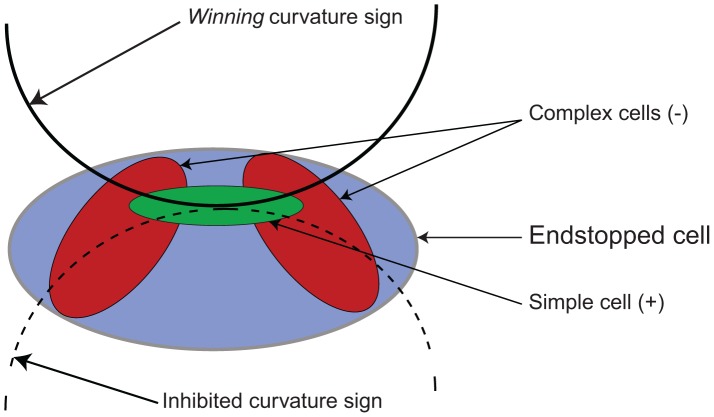
Model endstopped cell selective for curvature sign.

Apart from the degree of curvature, an additional contour characteristic that V2 cells seem to encode is the sign of curvature [28], [50]. Through the local information available to endstopping we may compute the sign of curvature. Here, in contrast to the curvature model cells, each displaced complex cell has a different orientation to the simple cell, and the two model complex cells are oriented at opposite signs (e.g. 45 and 135 for the 0 model endstopped neurons) (Figure 3). A hint regarding this concept was first proposed by [30], which is extended here to all orientations and used on curvatures.

For one sign of curvature, a curve excites the model simple excitatory cell at the center but curves falling into the region of the model complex inhibitory cells reduces the response of the model endstopped cell. A similar curve of the opposite sign passes only through the excitatory region (model simple cell), the curve having no inhibition effect (or a very low inhibition) on the overall response of the model endstopped cell since it is not, or is barely, falling on the model complex cell receptive fields (Figure 3).

Two types of model sign cells are used. These different signs are obtained by changing the order of the displaced subtracted neurons.


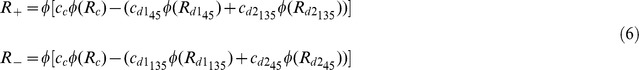


where 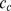, 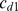 and 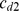 are the gains for the center and displaced cells as before. 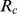, 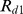 and 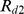 are the responses of center and displaced cells. The difference here is that the displaced cells are at different orientations of the preferred center simple cell, for the positive sign model endstopped neuron, the displaced model complex neuron *d1* is at 45, while the model complex component *d2* is at 135. For the negative sign model endstopped cell, the order is the opposite. For best results, these model cells required larger receptive field overlap than their degree of curvature endstopped model cells counterpart (see methods).

#### Model local curvature cells

This type of cell is the result of the combination of the responses from the two types of model endstoped cells (degree and sign of curvature), e.g. a model curvature cell that is selective for broad curvatures whose sign is positive as opposed to a model cell also selective for broad curvatures whose sign is negative. Through this neural convergence of model endstopped cells discriminative to the degree of curvature and the ones to the sign of curvature, we obtain twice the number of curvature classes. For example, if we have four types of model endstopped cells, through the use of the sign of curvature of those cells we obtain eight curvature classes.


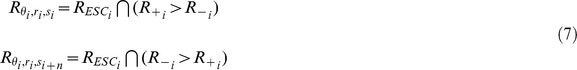


where 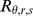 denotes the response of a neuron tuned to angle 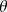, curvature *r* and sign *s*. *n* is the number of model endstopped cell types, 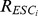 is the response of the model endstopped cell *i* and 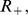 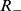 are the responses of the model sign selective endstopped neurons. In the realization of our model *i* = {1, 2, 3, 4} and *n* = 4 (see Material and Methods). This equation is read like: If the value of 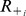 is greater than 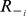, 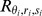 has the same value as the model curvature endstopped cell, otherwise, 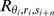 contains that value and 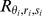 is 0. For the case where the response from endstopped cells is small, a high response from a model orientation simple cell means the contour is a straight line, so its curvature is set to 0. 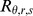 is computed at each location.

#### Model shape cells

V4 cells are quite sensitive to shape and less sensitive to spatial position [51]. Experiments in area V4 [52] and TEO [53], [54] of the macaque monkey seem to point to a strategy of recognition of objects by parts. In the case of V4 and TEO, those parts would be local curvatures [52], [54]–[56]. The response to a shape could correspond to the response of the local curvatures of the object. In TEO, some components of local curvatures excite the neuron, and others inhibit its response [54].

Neurons in areas V4 and TEO share similar characteristics regarding shape analysis [54], [56] and selectivity [57]. Although similar, TEO neurons show a higher degree of complexity than V4 neurons [54]. Our model shape neurons mimic that curvature by parts representation of shapes and silhouettes but are slightly more complex than just the curvature
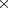
angular position coding proposed by [56] for V4 neurons since they are not only selective to curvatures at angular positions but also to the distance of the curvature element to the center of the shape. This conveys more information regarding the contour element. A shape would be different if the curvature is far away from the shape center or near the shape center even though its angular position is the same. We thus make use of both components to better describe the position of the curvature element than just one of them (angular position) as proposed in [56].

Our model shape cells integrate the responses from a population of model local curvature neurons to encode a shape. The proposed response of a model's shape neuron at location *x* is:


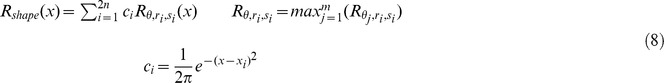


where 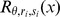 denotes the response of a model local curvature cell tuned to angle 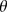, curvature *r* and sign *s* at location *x*, and 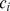 is a gaussian weight centered at 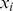 (*x* and 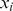 are in polar coordinates). *max* selects the maximum reponse from the local curvature over all angles, since the importance is on the responses to curvatures from curvature neurons, not their orientation at this level of the architecture. A model shape neuron will respond to a shape, and depending on how close the stimulus is to its selectivity (controlled through 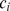 - see Materials and Methods), its response will be stronger or weaker. Total response of a shape neuron is the summation over all *p* locations:


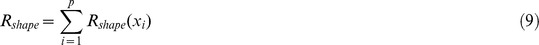


### Response of a model shape neuron in curvature space

**Figure 4:**
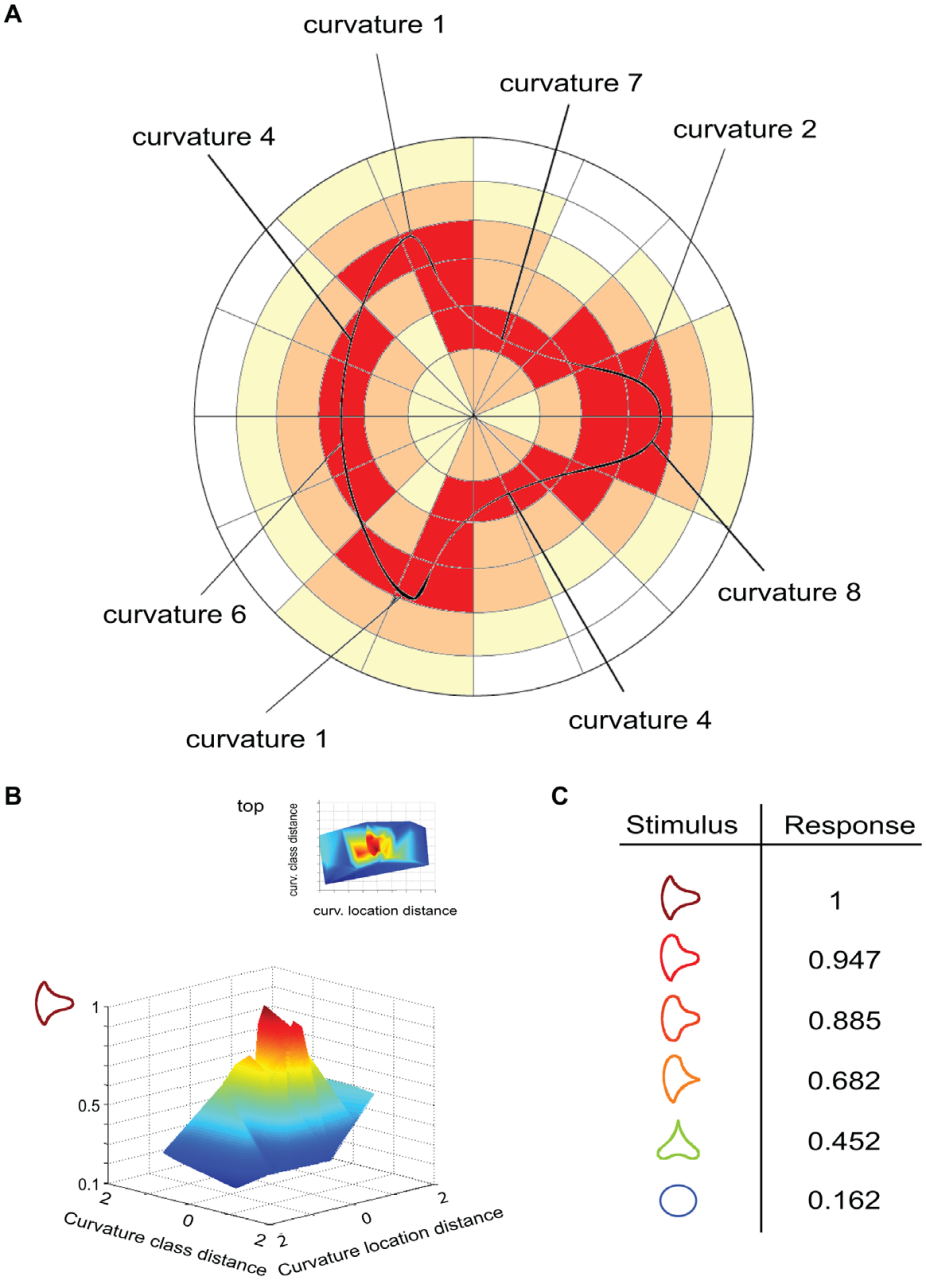
Shape-selective neuron. (A) Shape-selective neurons respond to different curvatures at different positions. The response is maximal when those curvatures are present at their selective positions (red). If they are in nearby positions the neuron provides some response as well (orange and yellow). (B) Shape-selective neuron tuning profile for location and curvature. (C) Shape neuron response to different stimuli, maximum response is to the stimulus at the top (value 1).

The model shape neuron of Figure 4A has a response depending on how close the stimulus is to its curvature-by-parts selectivity (Figure 4A). In the figure, the model neuron is selective to a sharp curvature at the top left. This neuron would respond maximally when that feature is present at that specific location, but it would respond also to a broader curvature at that location with a lower value and would have a small response to a very broad curvature or a straight line.

Model shape neurons exhibit band-pass tuning for curvature information. Their responses achieve a peak at a specific curvature, then decay providing a decreasing response for curvature values of increasing distance. No response is provided for curvatures very far from the optimal. The model shape neuron in this example is then selective for those model endstopped neurons that respond strongly to sharp curvatures at that position. Since a model endstopped neuron with a high response to a sharp curvature has also some response to a slightly broader type of curvature, model shape neurons will not provide a binary response but a range or responses depending on the distance between curvatures in curvature space (Figure 4B,C).

### Response of a model shape neuron based on curvature locations

Features (curvatures) comprising the model shape neuron are weighted with respect to a factor 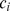 (Equation 8) depending on how close the desired curvature is to the desired position (Figure 4A). Continuing with the example of a neuron selective for a sharp curvature at the top left, this model neuron will have a high response to any stimuli that contain such sharp curvature at that position, but some response will still be elicited in a nearby position, e.g. a sharp curvature at the top mid-left, but no response will be obtained for a sharp curvature present at far away positions (e.g. the sharp curvature is at the bottom) (Figure 4B).

The curvatures that fall into the preferred cell's positions are considered in their full value (red in Figure 4A), but if they fall close, they are weighted in a Gaussian manner depending on how far from the preferred position they are (orange and yellow in Figure 4A).This is encoded using polar coordinates [52], that is, the radial distance to the center of the model shape neuron and its angular position.

#### Representational adequacy

In the words of Pasupathy and Connor [52]: *The population code for shape has to accomodate the virtual infinity of possible objects as well as the variability of a given object's retinal image*. Our model shape neuron has the capability of representing that virtual infinity of objects: If we consider that our stimuli are within 400
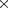
400 pixel images, for the bin size selection used in the experiments below (see Material and Methods) this gives a total of 1,800 possible curvature parts inside a model shape neuron receptive field. In the case of only 8 curvature classes, when we consider any possible combination of curvature/location, our model can represent a maximum of 14,400 (approximately 10 to the power of 86400) possible configurations of stimuli. In practice, one might take into account Gestalt properties such as continuity, proximity and others, and that number can be reduced to reflect only realizable configurations. The point here is that this representation is sufficiently rich to enable coding of a wide variety of shapes and task knowledge or learning through developmental experience will help determine the relevant subset for a given task domain.

### Comparison with biological neurons from area V4

Here we compare the performance of the model shape neurons with neurons in area V4 of the macaque's visual cortex from the same study on which our shape cells are based. For most cells in area V4 of the macaque, shapes evoking strongest responses are characterized by a consistent type of boundary configuration at a specific position within the stimulus [56]. We show that this behavior is compatible with the model shape-selective neurons constructed as explained previously.

Pasupathy and Connor [56] recorded the responses of 109 neurons to 366 different shapes. Each cell in the sample responded to a variety of very different shapes. No cell displayed a response pattern that could be characterized in terms of a single type of global shape. However, for most cells the effective stimuli showed some degree of shape consistency at one position. In other words, these cells were tuned for boundary configuration in one part of the shape.

In order to demonstrate the plausibility of our shape neurons and the hypothesis that curvature and shape may be encoded through endstopping, we study the behavior of the model shape neurons by comparing their responses against real neuron responses. We compared the responses from 75 - those cells where the shape consistency was more clear (see Material and Methods) - out of the 109 neurons recorded by Pasupathy and Connor's group. Data from real neurons to achieve this set of experiments was kindly provided by Dr. Anitha Pasupathy.

**Figure 5:**
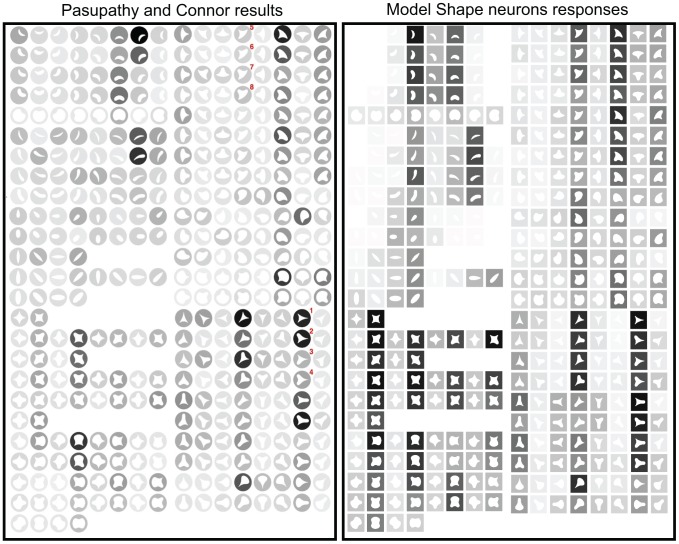
Comparison to **Figure 2** of [**56**]. Cells responses are on the left (
© 2001 The American Physiological Society, reproduced with permission) and their respective model responses are on right.

We first compared the responses from our shape-selective neurons with the four examples from [56]. We start with Figure 2 from [56] (our Figure 5). Real V4 neuron responses are on the left (stimuli within circles), our model shape neuron equivalent responses are on the right (stimuli within squares). Each row on both cases contains stimuli consisting of 2 shapes (one after the other) rotated in steps of 45. This is the stimulus set used by [56]. Each stimulus is represented by a white icon drawn within a circle (Pasupathy and Connor's results) or within a square (model shape neuron responses) representing the unit receptive field. The darker the background behind the icon, the higher the response of the neuron is to that shape, this applies both to Pasupathy and Connor's neuron recording and our model shape neuron.

For the cell in Figure 5, stimuli with a sharp convex angle at the bottom left were particularly effective (e.g. stimuli 1 and 2 in the middle column, bottom block; these stimuli are labeled with superscript numbers). Stimuli with a medium convex curve evoked moderate responses (e.g., stimuli 3 and 4). Thus this cell appears to encode information about the bottom left boundary region, responding well to sharp convexity at this location and poorly to broad convexity or concavity. Based on the response of this cell to the stimuli, this neuron was selective to a sharp convexity at the bottom left and a concavity adjacent to it (at the bottom). A first examination shows that the responses of the model's shape neurons are very similar to those of real cells. Our shape-selective neurons respond strongly to a sharp convexity at the botton left and a concavity at the bottom as well. If the curvature adjacent to the sharp convexity at the bottom left is convex, real cell responses are much weaker, our shape-selective neurons show also weaker responses as well but not as weak as for real cells. This additional weakness might be due to local inhibitory mechanisms (local competition) which are not presently included in the model.

**Figure 6:**
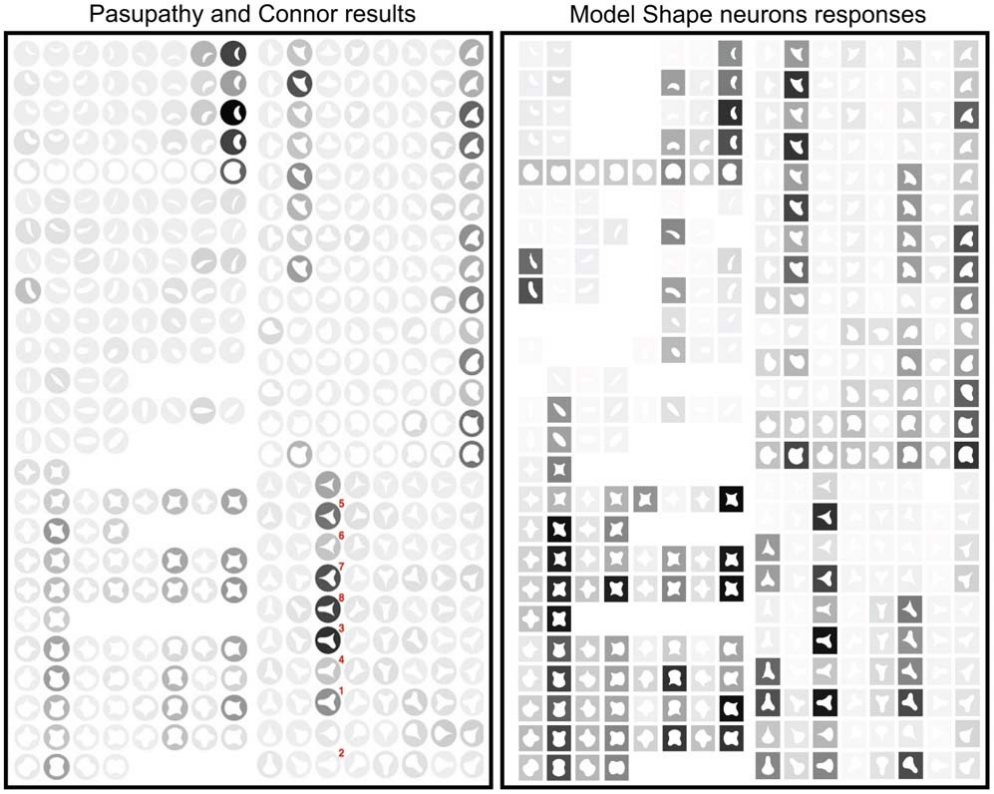
Comparison to **Figure 4** of [**56**]. Cells responses are on the left (
© 2001 The American Physiological Society, reproduced with permission) and their respective model responses are on right.

Another example provided by Pasupathy and Connor is on Figure 4 of their article (Replicated in Figure 6, right). This cell was sensitive to boundary configuration on the right side of the object, responding best to concave curvature at that position. This is exemplified by stimuli 1 and 2; stimulus 1, with a concavity at the right, evoked a stronger response. Stimulus 2 is almost identical, but with a convexity at the right, and it evoked no response. The cell also appears to be tuned for sharper convexities at the counter-clockwise-adjacent position and medium convexities at the clockwise-adjacent position. Pasupathy and Connor note that this is shown by stimulus 3 providing a strong response, while for stimulus 4, its response is weak (opposite combination: sharp curvature clockwise and medium curvature counter-clockwise). The results for the model in this case are almost equal for these stimuli as well as the other cases mentioned in [56]: compare shapes 5 and 6, and 7 and 8. As previously, there are some small differences, the model providing stronger responses than the real cells for a few stimuli.

**Figure 7:**
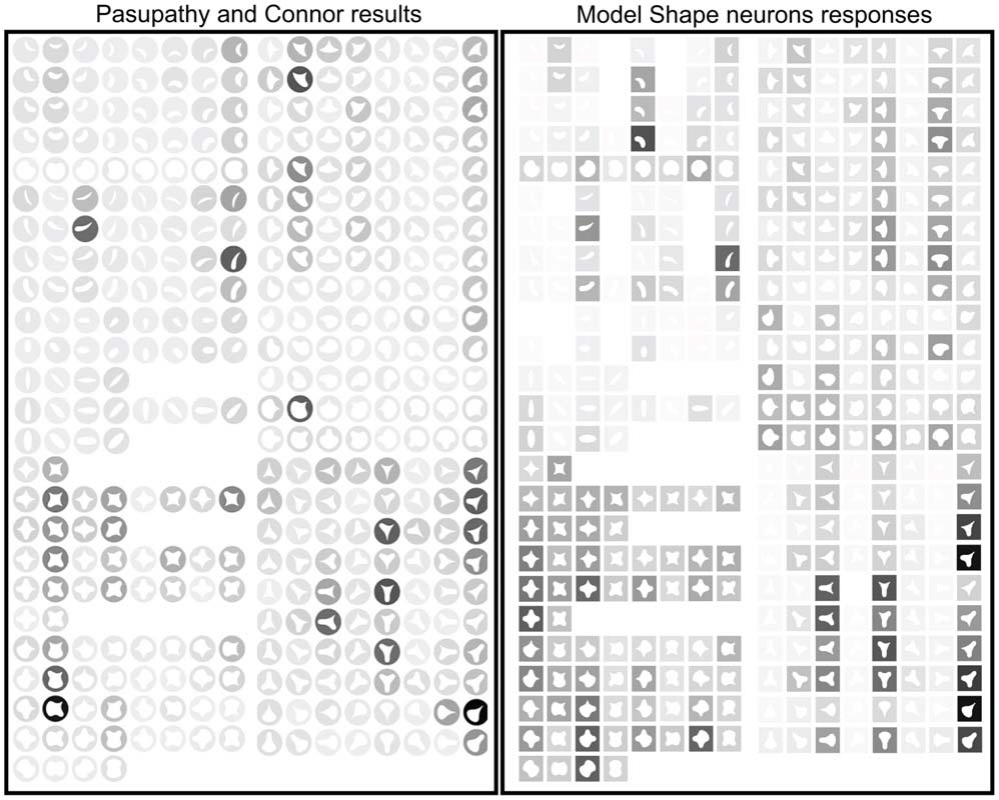
Comparison to **Figure 5** of [**56**]. Cells responses are on the left (
© 2001 The American Physiological Society, reproduced with permission) and their respective model responses are on right.

**Figure 8:**
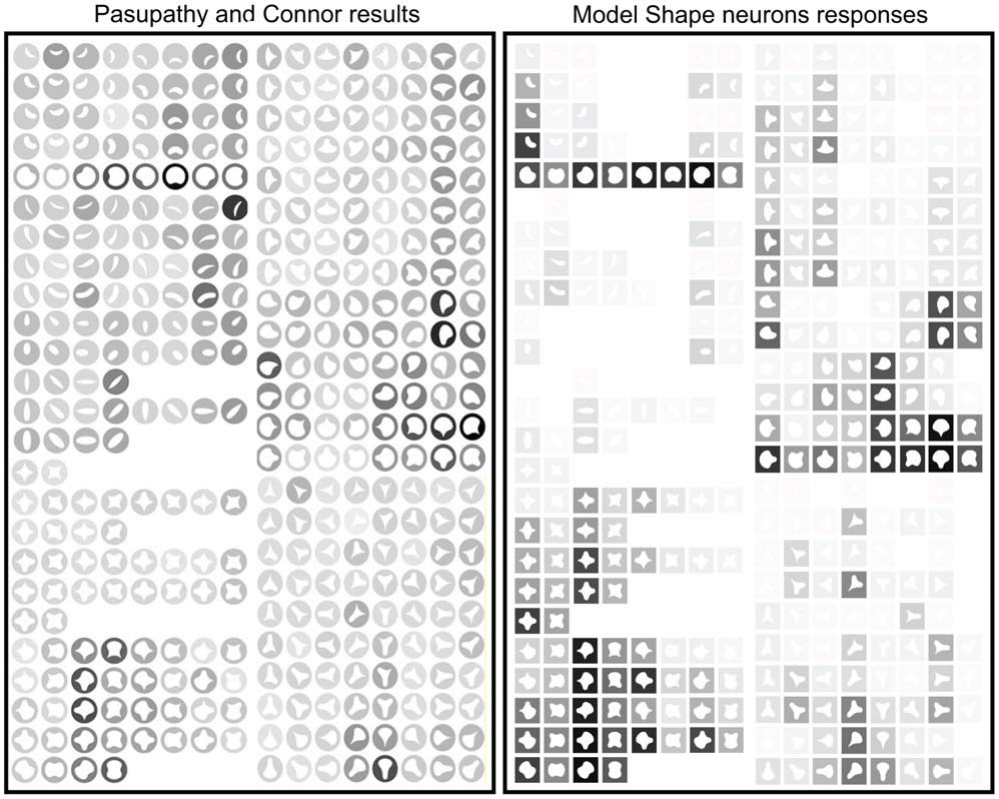
Comparison to **Figure 8** of [**56**]. Cells responses are on the left (
© 2001 The American Physiological Society, reproduced with permission) and their respective model responses are on right.

Figure 7 shows the comparison between one of our model shape neurons with the neuron corresponding to Figure 5 from [56]. This neuron was sensitive to a sharp convexity at the top right flanked by a concavity on one side or the other. A first examination shows that the responses of the model's shape neurons are very similar to those of real cells. As it is the case for Figure 8 of that same article, that cell was selective for broad convex curvature at the top. Their results are replicated here in Figure 8.

We compared the responses of 75 of our model shape neurons with 75 V4 cells. The comparison consisted in computing the absolute difference between the normalized responses of each model shape neuron and that of a real V4 neuron averaged over the 366 stimuli:


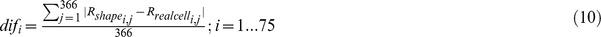



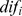
 is the absolute difference between each model shape neuron's response and the response from the real neuron. 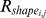 corresponds to the response of the *i-th* model shape neuron to the *j-th stimulus* and 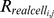 is the response of its real neuron counterpart to the same stimulus. For each cell, mean and standard deviation were computed and results will be provided next as error percentages, that is, mean difference between our model shape neurons and real cells.

**Figure 9:**
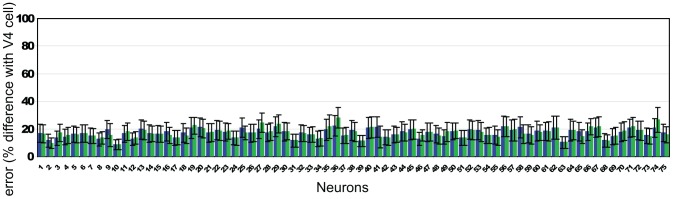
Difference between the model's Shape-selective neurons and 75 real cells responses from area V4.

The results for all the 75 cells considered in this study are shown in Figure 9 for two conditions: model neuron responses using the curvature parts with respect to the center of the neuron (blue bars) and model shape neuron responses with respect the centroid of the shape (green bars). Note that the stimuli from [56] are not always at the receptive field center. We did not find a significant difference between using curvature parts with respect to the center of the model neurons or the centroid of the object.

For both cases we can see that there are only a few model shape neurons with over 20% error, most of the differences between the model and that of real cells fall in the range 10–20%. Average error for all model shape neurons was 16.95% for the center of the model neuron (*stdev* = 12.61) and almost the same when using the centroid of the shape (*error* = 16.98%, *stdev* = 12.25). This shows that even for such a large number of neurons the model performs well and the difference between the response of the model shape-selective neurons and that of real cells is small. In direct comparison with the only other work to compare performance to this dataset of neural responses, our method significantly outperforms [25].

## Discussion

We have presented a model of 2D shape representation - 2DSIL - that follows the structure and behavior of the visual cortex. Building on past conjectures that one of the functional roles of endstopped cells may be to aid in shape analysis [28], [47], [55], we set out to define a biologically plausible computational model of shape representation. Here, we tested this hypothesis and have shown how a hierarchy starting from basic simple neurons, that combine into complex neurons and further endstopped neurons provide local curvature neurons that are selective for shape stimuli.

The main element in this architecture is that of the model shape-selective neuron, that represents curvature parts in a curvature
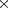
position (radial and angular) domain. The possible number of shapes that may be represented by our model shape neurons is very large, considering the limited types of neurons at each level of the architecture. Even though the primate visual system and our model have the capability to represent a virtual infinity of shapes, the way to handle the large but finite number of shapes in our world may be achieved through learning, selecting those configurations of curvatures relevant to recognize the shapes around us based on our visual experiences. Since the representation has the capability to represent any shape, a new shape can be easily incorporated into the system. The model supports a recognition by parts strategy, in which the parts are curvature values at different positions, as suggested also by Connor's group [54]. We have compared the response of our model shape neurons with 75 real neurons from [56]. The results obtained by the model are very similar to those of the neurons, and accomplished without any learning or classifier method.

Our model local curvature neurons do not provide an exact value of curvature but can discriminate among degrees of curvature (e.g. 4 in Figure 2C). This was done using a starting point where V1 is composed of neurons of different sizes. Through the use of different neuronal sizes and the integration of model simple neurons into model complex neurons we obtained model endstopped neurons able to discriminate between degrees of curvature, from very sharp to very broad (Figure 2C). It is important to note as well that these neurons do not provide a binary response for a given curve; model local curvature neurons provide a band-pass curvature filtering, with the highest response to the selective curvature and a decaying response that is inversely proportional to the curvature distances in curvature space. The response of model endstopped and curvature neurons over a range of curvatures have a Gaussian shape (Figure 2C), as well as a model shape neuron (Figure 4B). There is no maximum selection from the responses from early areas, so, no information is lost when ascending the hierarchy in a feedforward direction. However, there is a max selection computation at the last stage of the hierarchy, the shape cells, where it no longer affects further decisions, in keeping with Marr's Principle of Least Commitment [27]. We consider that any attentive selection, filtering or bias [58]–[61] in such a hierarchy would occur top-down and leave that for future work. Interestingly, our model of sign endstopped neurons could provide a foundation to deal with the border-ownership problem. Sign endstopped neurons could represent opponent channels [62], and this combined with feedback modulation through a model of attention (e.g. [58]) would further support a model such as the one presented by [33] on border ownership.

Our model may be considered as a major extension of the works [9] and [28], [30]. In a similar work, Serre, Cadieu and colleagues construct a hierarchical representation with a first layer computing oriented edge responses. This is followed by a maximum response selection layer that feeds a pooling stage that groups spatial piece-wise linear elements. This strategy - borrowed from Fukushima's NeoCognitron [9] - is repeated for each layer of the hierarchy. Curved lines are thus approximated by linear pieces and there is no direct computation of curvature of any form. Another related model, based on excitatory connections is the one proposed by Amit [20]. One important difference (among others) between our model and these types of models is that we use inhibition for curvature representation through endstopping instead of purely excitatory components. Inhibitory flankers as proposed in our model have been strongly supported by neurophysiological studies [39], [44]–[46], [48], [49] and since our goal is to test the computational embodiment of these neurophysiological results, this necessarily figures prominently in our model. It is an aspect that is considered of great importance by neuroscientists [46], and surprisingly has been neglected in models to date.

Given that it seems accepted that the visual system computes increasingly abstract quantities as a signal ascends the visual processing hierarchy, are those quantities computed by applying the same computation and thus neural convergence alone suffices to achieve abstraction, or, is it truly necessary to include more sophisticated computations layer by layer? This is not easy to answer in the general case. However, we can point to one important instance that supports the latter position. In our previous work where we look at motion processing [63], we found that simple neural convergence did not suffice. We needed to include a layer of neurons selective to the spatial derivative of velocity, a much more complex construct. This is supported by neurophysiology in monkey [64], [65] and by our own fMRI human studies [66]. Similarly, for shape representation, although our approach is also based on a hierarchical set of computations, we deploy different processes at each layer, not simply repetitions of the same process. Those different processes are intended to reflect the reality of the different neural computations in the visual cortex. Our approach is distinct in that we perform a direct computation of curvature and the sign of curvature. We develop that computation using well documented neural computation types that include not only oriented simple cells and complex cells (as the pooling layer of others is intended to capture) but also endstopped cells, curvature cells, and curvature sign cells. These naturally provide a sufficient basis for the definition of shape cells, a basis that not only mirrors neurophysiological reality of the visual cortex better, but also provides a richer substrate for shape definition than piecewise linear components. This is the first model of shape representation (to the best of our knowledge) to include aforementioned cells in intermediate layers departing from the near universal previous use of Fukushima's *S* and *C* types of cells.

The role of learning from examples also differs between our work and those mentioned. Although a statistical learning approach such as that employed by Serre, Cadieu and colleagues for all of the layers of their processing hierarchy except for the first, is valuable when there is no other option, we show that in the case of the successive representations, namely those computed by endstopped and curvature cells, there is now sufficient knowledge to directly model these cells and to do so with a significantly high degree of fidelity. Learning is not required if the appropriate representations are selected in the first place.

Although this paper does not address object recognition directly, it may provide important contributions to elements that may advance the state-of-the-art. In a previous paper [34], we connected the 2DSIL representation to a recognition system and compared its performance in object recognition tasks with several other systems including benchmark systems. Our system performed well beating other systems in several categories while maintaining comparable performance in others. Following previous authors such as Zucker and Marr, we advocate that deeper understanding of visual processes in humans and non-human primates can lead to important advancements in perceptual theories and computational systems.

With the model introduced in this paper we follow the steps of early theories of vision [9], [26], [67] and propose how to – following the philosophy of those influential works – take modeling to a next stage by incorporating new intermediate layer computations hoping future works will continue building on these hierarchies aimed at modeling the visual cortex.

## Materials and Methods

We used the same stimuli created for [52], [56]. In order to construct the stimuli, a Matlab program was provided by Dr. Anitha Pasupathy. The stimuli were constructed combining convex and concave boundary elements to form closed shapes. Boundary elements include sharp convex angles, and medium and high convex and concave curvatures. The combination of these boundary elements gave rise to 49 different stimuli. Stimuli were composed of white edges against a black background, the inside was black as well but it is shown in our figures (Figures 5, 6, 7, and 8) as white-filled for illustration purposes. For the experiment, stimuli were those 49 shapes but rotated to 8 orientations (some only 2 or 4 due to redundancies) in 45 increments to give a total of 366 different shapes. Stimuli are shown in Figures 5, 6, 7, and 8.

Experiments were run on Matlab in a Mac G5 PowerPC. The input to the model is a gray-value image. Images used are 400
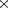
400 pixels, a shape would span 300
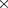
300 pixels and correspond to the stimuli used in the aforementioned study. For our experiments, we used 12 orientations (0, 15, 30, 45, 60, 75, 90, 105, 120, 135, 150, 165) and 4 different sizes for model simple cells, this gives a total of 48 types. Size of V1 model simple neurons are 40, 60, 88 and 120 pixels, their corresponding values for AR are 0.7, 1.4, 2.15 and 3 respectively, WR is 2.5 for all model neurons.

For the integration into model endstopped neurons, the values of gain *c* (Equation 4) for displaced neurons were from the smaller to the larger cell: 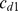 = 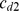 = {1.5, 1.25, 1, 3}, 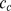 = 1 for all centre cells. For the chosen parameters, cells respond (90% of their maximum value) to the following ranges of curvature radius: 6 to 11, 25 to 52, 48 to 77 and 140 to 301 pixels. Refer also to Figure 2C for an example on how the selection of these parameters (size, AR, WR and gain) affect neuronal curvature selectivity. The parameters for the rectification function (Equation 5) were 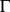 = 0.01 and 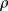 is the maximum response of the set of neurons for a given scale divided by 8.5, a factor that provided a good normalization approximation for this sigmoidal saturation function. The displacement values for model endstopped neurons selective to degrees of curvature was 1/2 the size of the simple neuron component along its preferred orientation. Displacements for the model sign endstopped neurons were from smaller to larger: 1/5 the size, 1/4 the size, 1/4 the size and 2/5 the size along the orientations stated in Equation 6. The 4 types of model endstopped neurons and the curvature direction selective neurons lead to eight curvatures. In order to obtain the aforementioned parameter values, a program designed to evaluate different parameter values was created. The target of this program was to obtain values that would provide neurons able to separate different degrees of curvature, providing a graph such as the one shown in Figure 2C.

**Figure 10:**
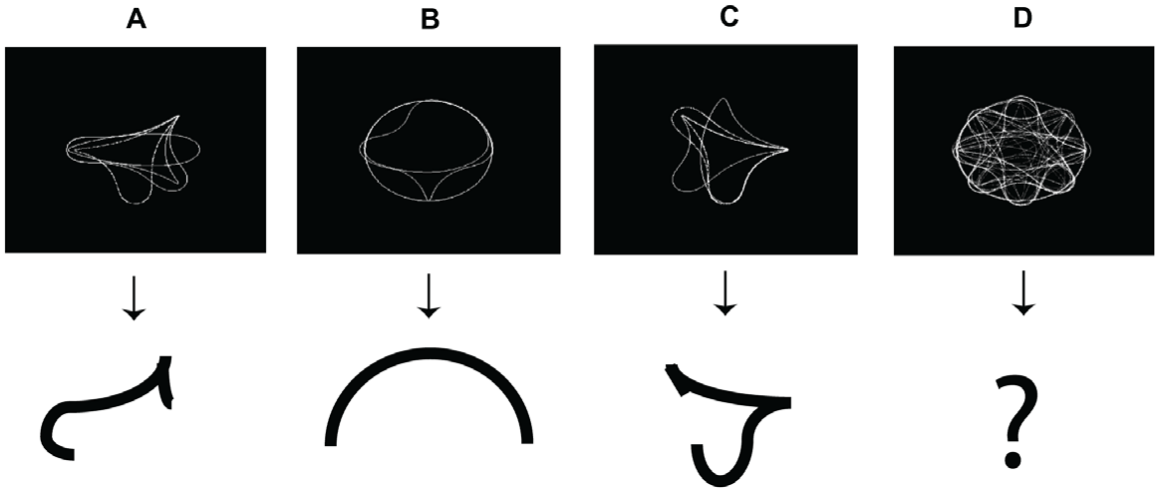
How the features for *isolating* a Shape neuron are obtained. See text.

Neuron responses were provided by Dr. Anitha Pasupathy for the comparison with model shape neuron responses. In their influential study [56], the results from 109 neurons are reported for 366 different stimuli. We compared with 75 out of those 109 neurons, the reason for this as well as the detailed process are explained next. Due to the enormous range of shape representation of the model, we needed to select (or *isolate* in neurophysiological terms) a subset of model shape neurons that would correspond to their 109 subset of V4 biological counterparts recorded in [56]. In order to do this, we created new stimulus images and stored their model shape representation. The way these stimuli were created was by superimposing the stimuli for which the biological neuronal responses were on the 70% maximum percentile (e.g. Figure 10A). This simple process would give us an insight on the selectivity of the 109 biological neurons and is similar to the way [56] analyzes the selectivity of 4 neurons (Figures 2, 4, 5 and 8 on that work). That is, we consider the stimuli that maximize the neuron responses to reach the conclusion that a neuron is selective to some type of curvature at a specified position, e.g. in Figure 10A it is clear that this biological neuron is selective for a sharp curvature at the top-right, flanked by a broad concavity that ends in a medium convexity on the left side of the stimulus. Then, this image would be modified such as to only keep the relevant curvatures. This is the stimulus used to isolate our model shape neurons. This would also be the stimulus for which the model shape neuron response is maximum.

We repeated this process for the 109 biological neurons, but 34 of them failed to provide any clear insight on their selectivity using the present process (e.g. Figure 10D). On the other hand, the other 75 provided a very clear picture on their selectivities (Figure 10A–C). We then stored the representation (Figure 4A) of each shape model neuron for the stimuli created the way explained above. The weights 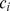 (Equation 8) are derived from the responses from the eight curvature classes model neurons at their different positions. Model shape neuron's receptive fields were organized into angular-radial bins (Figure 4A) of 10 pixels for radial values and 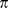/45 for angular values. A smaller bin size did not provide significantly better results while having a much higher computational load.

For each one of the model shape neurons isolated this way, we recorded responses for each of the 366 stimuli in [56]. Each response is normalized in the 0–1 range using the maximum response for the created stimulus as explained before as the normalization factor. These normalized responses were compared to their biological counterparts (responses already normalized) and the absolute value of the difference was computed for each one of the 366 stimuli. Figure 9 shows the results of these averaged values with their corresponding standard deviations for each neuron.

## References

[1] Ramón y Cajal S (1888) Sobre las fibras nerviosas de la capa molecular del cerebelo. Revista Trimestral de Histología Normal y Patologica 1: 33–49.

[2] Ramón y Cajal S (1894) The croonian lecture: La fine structure des centres nerveux. Royal Society of London Proceedings Series I 55: 444–468.

[3] Ramón y Cajal S (1904) Variaciones morfólogicas, normales y patológicas del retículo neurofibrilar. Trabajos del Laboratorio de Investigaciones Biólogicas Madrid 3: 9–15.

[4] Jones E (2007) Neuroanatomy: Cajal and after cajal. Brain Research Reviews 55: 248–255. 17659350 10.1016/j.brainresrev.2007.06.001

[5] Grossberg S (1968) Some nonlinear networks capable of learning a spatial pattern of arbitrary complexity. Proceedings of the National Academy of Sciences of the United States of America 59: 368–72. 16591608 10.1073/pnas.59.2.368 PMC224680

[6] Grossberg S (1970) Neural pattern discrimination. Journal of Theoretical Biology 27: 291–337. 5452497 10.1016/0022-5193(70)90143-8

[7] Grossberg S (1971) Pavlovian pattern learning by nonlinear neural networks. Proceedings of the National Academy of Sciences of the United States of America 68: 828–31. 4323791 10.1073/pnas.68.4.828 PMC389053

[8] Grossberg S (1975) A neural model of attention, reinforcement and discrimination learning. International Review of Neurobiology 18: 263–327. 1107246 10.1016/s0074-7742(08)60037-9

[9] Fukushima K (1980) Neocognitron: a self organizing neural network model for a mechanism of pattern recognition unaffected by shift in position. Biological Cybernetics 36: 193–202. 7370364 10.1007/BF00344251

[10] Fukushima K, Miyake S, Ito T (1983) Neocognitron: A neural network model for a mechanism of visual patter recognition. IEEE Transactions on Systems, Man and Cybernetics 13.

[11] Rumelhart D, Mcclelland J (1986) Parallel Distributed Processing: Explorations in the Microstructure of Cognition. MIT Press. 10.1111/cogs.12148 25087578

[12] Lecun Y, Boser B, Denker J, Henderson D, Howard R, et al (1989) Backpropagation applied to handwritten zip code recognition. Neural Computation 1: 541–551.

[13] Lecun Y, Bottou L, Bengio Y, Haffner P (1998) Gradient-based learning applied to document recognition. Proceedings of the IEEE 86: 2278–2324.

[14] Wallis G, Rolls E (1997) Invariant face and object recognition in the visual system. Progress in Neurobiology 51: 167–194. 9247963 10.1016/s0301-0082(96)00054-8

[15] Riesenhuber M, Poggio T (1999) Hierarchical models of object recognition in cortex. Nature Neuroscience 2: 1019–1025. 10526343 10.1038/14819

[16] Riesenhuber M, Poggio T (2000) Models of object recognition. Nature Neuroscience 3 Suppl: 1199–1204. 11127838 10.1038/81479

[17] Riesenhuber M, Poggio T (2002) Neural mechanisms of object recognition. Current Opinion in Neurobiology 12: 162–168. 12015232 10.1016/s0959-4388(02)00304-5

[18] Serre T, Wolf L, Poggio T (2005) Object recognition with features inspired by visual cortex. IEEE Conference on Computer Vision and Pattern Recognition (CVPR) 994–1000.

[19] Serre T, Wolf L, Bileschi S, Riesenhuber M (2007) Robust object recognition with cortex-like mechanisms. IEEE Transactions on Pattern Analysis and Machine Intelligence 29: 411–426. 17224612 10.1109/TPAMI.2007.56

[20] Amit Y (2000) A neural network architecture for visual selection. Neural Computation 12: 1141–1164. 10905811 10.1162/089976600300015538

[21] Amit Y, Mascaro M (2003) An integrated network for invariant visual detection and recognition. Vision Research 93: 2073–2088. 10.1016/s0042-6989(03)00306-7 12842160

[22] Suzuki N, Hashimoto N, Kashimori Y, Zheng M, Kambara T (2004) A neural model of predictive recognition in form patway of visual cortex. BioSystems 76: 33–42. 15351128 10.1016/j.biosystems.2004.05.004

[23] Rao R, Ballard D (1997) Dynamic model of visual recognition predicts neural response properties in the visual cortex. Neural Computation 9: 721–763. 9161021 10.1162/neco.1997.9.4.721

[24] Rao R, Ballard D (1999) Predictive coding in the visual cortex: a functional interpretation of some extra-classical receptive-field effects. Nature Neuroscience 2: 79–87. 10195184 10.1038/4580

[25] Cadieu C, Kouch M, Connor C, Riesenhuber M, Poggio T (2007) A model of v4 shape selectivity and invariance. Journal of Neurlophysiology 3: 1733–1750. 10.1152/jn.01265.2006 17596412

[26] Marr D, Hildreth E (1980) Theory of edge detection. Proceedings of the Royal Society of London, series B, Biological Sciences 207: 187–217. 10.1098/rspb.1980.0020 6102765

[27] Marr D (1982) Vision: A Computational Investigation into the Human Representation and Processing of Visual Information. W.H. Freeman.

[28] Dobbins A, Zucker S, Cynader M (1987) Endstopped neurons in the visual cortex as a substrate for calculating curvature. Nature 329: 438–441. 3657960 10.1038/329438a0

[29] Heitger F, Rosenthaler L, von der Heydt R, Peterhans E, Kubler O (1992) Simulation of neural contour mechanisms: from simple to end-stopped cells. Vision Research 32: 963–81. 1604865 10.1016/0042-6989(92)90039-l

[30] Dobbins A (1992) Difference models of Visual Cortical neurons. Ph.D. thesis, Department of Electrical Engineering. McGill University.

[31] Heitger F, von der Heydt R (1993) A computational model of neural contour processing: figureground segregation and illusory contours. Proceedings of the IEEE International Conference on Computer Vision 32–40.

[32] Heitger F, von der Heydt R, Peterhans E, Rosenthaler L, Kübler O (1998) Simulation of neural contour mechanisms: representing anomalous contours. Image and Vision Computing 16: 409–423.

[33] Craft E, Schuetze H, Niebur E, von der Heydt R (2007) A neural model of figure-ground organization. Journal of Neurophysiology 97: 4310–4326. 17442769 10.1152/jn.00203.2007

[34] Rodríguez-Sánchez A, Tsotsos J (2011) The importance of intermediate representations for the modeling of 2 d shape detection: Endstopping and curvature tuned computations. IEEE Conference on Computer Vision and Pattern Recognition (CVPR) 4321–4326.

[35] Marcelja S (1980) Mathematical description of the responses of simple cortical cells. Journal of Optical Society of America 70: 1297–1300. 10.1364/josa.70.001297 7463179

[36] Hawken M, Parker A (1987) Spatial properties of neurons in the monkey striate cortex. Proceedings of the Royal Society of London, series B, Biological Sciences 231: 251–288. 10.1098/rspb.1987.0044 2889214

[37] Hubel D, Wiesel T (1959) Receptive fields of single neurones in the cat's striate cortex. The Journal of Physiology 148: 574–591. 14403679 10.1113/jphysiol.1959.sp006308 PMC1363130

[38] Hubel D, Wiesel T (1962) Receptive fields, binocular interaction and functional architecture in the cat's visual cortex. Journal of Physiology 160: 106–154. 14449617 10.1113/jphysiol.1962.sp006837 PMC1359523

[39] Hubel D, Wiesel T (1968) Receptive fields and functional architecture of monkey striate cortex. Journal of Physiology 195: 215–243. 4966457 10.1113/jphysiol.1968.sp008455 PMC1557912

[40] Spitzer H, Hochstein S (1985) A complex-cell receptive-field model. Journal of Neurophysiology 53: 1266–1286. 3998809 10.1152/jn.1985.53.5.1266

[41] Geman S (2006) Invariance and selectivity in the ventral visual pathway. Journal of Physiology Paris 100: 212–224. 10.1016/j.jphysparis.2007.01.001 17336506

[42] von der Heydt R, Peterhans E, Baumgartner G (1984) Illusory contours and cortical neuron responses. Science 224: 1260–1262. 6539501 10.1126/science.6539501

[43] Ito M, Komatsu H (2004) Representation of angles embedded within contour stimuli in area v2 of macaque monkeys. Journal of Neuroscience 24: 3313–3324. 15056711 10.1523/JNEUROSCI.4364-03.2004 PMC6730022

[44] Kato H, Bishop P, Orban G (1978) Hypercomplex and simple/complex cells classifications in cat striate cortex. Journal of Neurophysiology 1071–1095. 702188 10.1152/jn.1978.41.5.1071

[45] Orban G, Kato H, Bishop P (1979) Dimensions and properties of end-zone inhibitory areas of hypercomplex cells in cat striate cortex. Journal of Neurophysiology 42: 833–849. 430120 10.1152/jn.1979.42.3.833

[46] Willmore BD, Prenger RJ, Gallant JL (2010) Neural representation of natural images in visual area v2. The Journal of Neuroscience 30: 2102–14. 20147538 10.1523/JNEUROSCI.4099-09.2010 PMC2994536

[47] Dobbins A, Zucker S, Cynader M (1989) Endstopping and curvature. Vision Research 29: 1371–1387. 2635466 10.1016/0042-6989(89)90193-4

[48] Orban G, Kato H, Bishop P (1979) End-zone region in receptive fields of hypercomplex and other striate neurons in the cat. Journal of Neurophysiology 42: 818–832. 430119 10.1152/jn.1979.42.3.818

[49] Bishop P, Kato H, Orban G (1980) Direction-selective cells in complex family in cat striate cortex. Journal of Neurophysiology 43: 1266–1283. 7373366 10.1152/jn.1980.43.5.1266

[50] Hegde J, Van Essen DC (2000) Selectivity for complex shapes in primate visual area v2. The Journal of Neuroscience 20: 61–66. 10.1523/JNEUROSCI.20-05-j0001.2000 PMC6772904 10684908

[51] Gallant J, Braun J, Van Essen D (1993) Selectivity for polar, hyperbolic, and cartesian gratings in macaque visual cortex. Science 259: 100–103. 8418487 10.1126/science.8418487

[52] Pasupathy A, Connor C (2002) Population coding of shape in area v4. Nature Neuroscience 5: 1332–1338. 12426571 10.1038/nn972

[53] Tanaka K (1996) Inferotemporal cortex and object vision. Annual Review on Neuroscience 19: 109–139. 10.1146/annurev.ne.19.030196.000545 8833438

[54] Brincat S, Connor C (2004) Underlying principles of visual shape selectivity in posterior inferotemporal cortex. Nature Neuroscience 7: 880–886. 15235606 10.1038/nn1278

[55] Pasupathy A, Connor C (1999) Responses to contour features in macaque area v4. Journal of Neurophysiology 82: 2490–2502. 10561421 10.1152/jn.1999.82.5.2490

[56] Pasupathy A, Connor C (2001) Shape representation in area v4: Position-specific tuning for boundary conformation. Journal of Neurophysiology 86: 2505–2519. 11698538 10.1152/jn.2001.86.5.2505

[57] Boussaoud D, Desimone R, Ungerleider L (1991) Visual topography of area teo in the macaque. The Journal of Comparative Neurology 306: 554–575. 1712794 10.1002/cne.903060403

[58] Tsotsos J, Culhane S, Winky W, Lai Y, Davis N, et al (1995) Modeling visual attention via selective tuning. Artificial Intelligence 78: 507–545.

[59] Desimone R, Duncan J (1995) Neural mechanisms of selective visual attention. Annual Review on Neuroscience 18: 193–222. 10.1146/annurev.ne.18.030195.001205 7605061

[60] Rodríguez-Sánchez A, Simine E, Tsotsos J (2007) Attention and visual search. International Journal of Neural Systems 17: 275–288. 17696292 10.1142/S0129065707001135

[61] Tsotsos JK (2011) A Computational Perspective on Visual Attention. The MIT Press.

[62] Zhou H, Friedman HS, von der Heydt R (2000) Coding of border ownership in monkey visual cortex. The Journal of Neuroscience 20: 6594–6611. 10964965 10.1523/JNEUROSCI.20-17-06594.2000 PMC4784717

[63] Tsotsos J, Liu Y, Martíinez-Trujillo J, Pomplun M, Simine E, et al (2005) Attending to visual motion. Computer Vision and Image Understanding 100: 3–43.

[64] Treue S, Andersen R (1996) Neural responses to velocity gradients in macaque cortical area mt. Visual Neuroscience 13: 797–804. 8870234 10.1017/s095252380000866x

[65] Meese T, Anderson S (2002) Spiral mechanisms are required to account for summation of complex motion components. Vision Research 42: 1073–1080. 11997046 10.1016/s0042-6989(02)00058-5

[66] Martínez-Trujillo J, Tsotsos J, Simine E, Pomplun M, Wildes R, et al (2005) Selectivity for speed gradients in human area mt/v5. Neuroreport 16: 435–438. 15770147 10.1097/00001756-200504040-00004

[67] Zucker SW (1981) Computer vision and human perception: An essay on the discovery of constraints. Proceedings of the International Conference on Artificial Intelligence 1102–1116.

